# A refined protocol for the large-scale production of high-quality cerebral organoids

**DOI:** 10.1007/s00702-026-03216-x

**Published:** 2026-07-03

**Authors:** Tatyana Prokhorova, Sonja Simone Hohmann, Alessia Fabozzi, Silvia Saltarelli, Vyacheslav Akimov, Cristine Marie Yde Ohki, Rhiannon V. McNeill, Christian Tvedesøe, Blagoy Blagoev, Sarah Kittel-Schneider, Peter Schneider-Kamp, Lukasz Smigielski, Jing Qin, Birgit Debrabant, Edna Grünblatt, Tanja Maria Michel

**Affiliations:** 1 Department of Psychiatry, Department of Clinical Research, University of Southern Denmark, Odense, Denmark; 2 Psychiatry in the Region of Southern Denmark, Odense University Hospital, Odense, Denmark; 3 Department of Biochemistry and Molecular Biology, Center for Experimental BioInformatics, University of Southern Denmark, Odense, Denmark; 4 Department of Child and Adolescent Psychiatry and Psychotherapy, Translational Molecular Psychiatry, Psychiatric University Hospital Zurich, University of Zurich, Wagistrasse 12, 8952 Schlieren, Switzerland; 5 Department of Psychiatry, Psychosomatics and Psychotherapy, University Hospital Würzburg, Würzburg, Germany; 6 Department of Psychiatry and Neurobehavioural Science, University College Cork, Cork, Ireland; 7 Department of Mathematics and Computer Science, University of Southern Denmark, Odense, Denmark; 8 Neuroscience Center Zurich, University of Zurich and the ETH Zurich, Winterthurerstrasse 11, 8057 Zurich, Switzerland; 9 Zurich Center for Integrative Human Physiology, University of Zurich, Winterthurerstrasse 11, 8057 Zurich, Switzerland; 10 Psychiatric and Psychotherapeutic Hospital, University Hospital Erlangen, Friedrich-Alexander University Erlangen-Nuremberg, Erlangen, Germany

**Keywords:** Cerebral organoids, iPSCs, Large-scale culture, Neuroepithelial formation, Organoid heterogeneity, Proteomics

## Abstract

Human pluripotent stem cell-derived cerebral organoids have emerged as a valuable tool for investigating early brain development and modeling neurodevelopmental disorders. However, considerable variation in both organoid size and cellular composition has been widely reported. Moreover, the large-scale production of organoids using conventional methods is a significant challenge, particularly considering that some stem cell lines exhibit variable efficiency in neuroepithelial formation. This heterogeneity of cerebral organoids can complicate experimental reproducibility and comparability in studies of brain development and disease mechanisms. Therefore, we developed a protocol using CERO 3D bioreactors, which enables scalable organoid production and improves size homogeneity compared to conventional approaches. In addition, we conducted a pilot study, utilizing MS-based proteomic analysis of single organoids from two different lines, aiming to explore whether size variability is associated with differences in protein expression within cerebral organoids. The organoids produced in the CERO 3D bioreactors reproducibly form neuroepithelial structures and express key neurodevelopmental markers. The presented protocol provides an efficient way of producing cerebral organoids, supporting reproducibility and high-throughput applications in neurodevelopmental research.

## Introduction

Cerebral organoids provide a three-dimensional model system for studying early human brain development and associated physiological and pathological processes (Lancaster et al. 2013; Eichmuller and Knoblich 2022). They are commonly generated from human pluripotent stem cells, including induced pluripotent stem cells (iPSCs), which retain the donor’s genetic background and therefore enable the investigation of individual-specific neurodevelopmental features.

Despite their broad applicability, cerebral organoid cultures are characterized by substantial variability in size, morphology, and cellular composition, even within the same iPSC line and experimental batch (Qian et al. 2018; Del Dosso et al. 2020; Hong et al. 2022; Dutan Polit et al. 2023). In addition, conventional plate- or shaker-based culture methods are labor-intensive, difficult to scale, and limited in throughput. These challenges complicate experimental reproducibility and comparability, particularly in studies requiring large numbers of organoids or systematic downstream analyses.

A further limitation arises during embryoid body (EB) formation, a critical early step in cerebral organoid generation. Typically, only 30–80% of EBs successfully form expanded neuroepithelium, an essential prerequisite for proper cerebral organoid development, and this efficiency varies considerably between iPSC lines (Lancaster et al. 2013; Lancaster and Knoblich 2014). Improving the robustness and efficiency of neuroepithelial formation is essential for optimizing cerebral organoid culture protocols.

To address these challenges, we developed a refined protocol for cerebral organoid generation using the CERO 3D benchtop bioreactor, a near-microgravity culture system that maintains organoids in continuous suspension through alternating rotational motion. This approach enhances nutrient distribution, reduces mechanical stress, and enables the scalable production of large numbers of organoids with improved size homogeneity compared with conventional culture methods.

Near-microgravity or rotating suspension bioreactor systems have previously been applied to a range of 3D cell culture models, including spheroids and organoid-like cultures, to improve nutrient distribution and scalability (Wuest et al. 2014; Altmaier et al. 2022; Stange et al. 2022). However, to our knowledge, a dedicated protocol for large-scale cerebral organoid production using the CERO 3D system has not been reported.

In addition to presenting this protocol, we performed an exploratory pilot analysis using mass spectrometry (MS)-based proteomics of individual organoids to assess whether differences in organoid size are associated with detectable differences in protein expression. Together, these data provide both a practical workflow for high-throughput cerebral organoid production and supporting evidence highlighting the importance of size control for downstream molecular analyses.

## Materials and methods

### Cell lines and cell culture

We included iPSC lines from 12 different individuals (listed in Table 1).

**Table 1:** Overview of iPSC lines used in this study

iPSC line number	iPSC line ID	Donor age (years)	Donor gender	iPSC quality control reference
1	MR-001	15	Male	Grossmann et al. (2021)
2	MR-010	9	Male	Grossmann et al. (2021)
3	MR-013	17	Male	Grossmann et al. (2021)
4	MR-014	13	Male	Grossmann et al. (2021)
5	KO-001	15	Male	Yde Ohki et al. (2021)
6	KO-005	16	Male	Yde Ohki et al. (2021)
7	KO-011	16	Male	Yde Ohki et al. (2021)
8	KO-015	15	Male	Yde Ohki et al. (2023)
9	SK55	39	Female	Palladino et al. (2018)
10	WLZ368	35	Female	Palladino et al. (2018)
11	AP2	21	Male	Kamand et al. (2020)
12	CT1	20	Male	Kamand et al. (2022)

iPSC lines were generated from plucked hair-derived keratinocytes, peripheral blood mononuclear cells (PBMCs) or skin fibroblasts using Sendai virus or non-integrating plasmids and characterised as described in references in Table 1. For clarity, we will refer to them as iPSC line 1, iPSC line 2 etc.

iPSCs were grown on plates (Corning) coated with hESC-qualified Matrigel (MG, Corning, 354277) in mTeSR+ media (STEMCELL Technologies, 100-0276). Cells were passaged using ReLeSR (STEMCELL Technologies, 100-0483).

### Cerebral organoid generation

Organoids were generated via intermediate EB formation and consecutive exposure to pre-defined neural induction media (STEMdiff™ Cerebral Organoid Kit, STEMCELL Technologies, 08570), using a previously published and modified protocol (Lancaster et al. 2013), as described below:

#### Embryoid body formation

Briefly, iPSCs were dissociated into a single cell suspension using Gentle Cell Dissociation Reagent (GCDR, STEMCELL Technologies, 100–0485), counted, and plated in seeding medium (SM) into AggreWell 800 plates (STEMCELL Technologies, 34811) coated with Anti-Adherence Rinsing Solution (AARS, STEMCELL Technologies, 07010) at a density of 3000 cells/microwell (day 0). SM was replaced by formation medium (FM) on day 1. Fresh FM was supplemented on days 2, 3, and 4. On day 5, FM was replaced by induction medium (IM). Fresh IM was supplied on days 6 and 7. On day 8, EBs were supplemented with MG and moved into expansion medium (EM) for further culture in plates on a shaker or in a CERO 3D bioreactor, as described below.

#### Organoid formation

For further culture in plates, eight-day-old EBs were embedded into MG droplets individually, essentially as described (Lancaster et al. 2013), and placed into six-well plates, pre-coated with AARS. This was referred to as organoid day 0. Plates were incubated on an orbital shaker for three days in a 37 °C incubator at 5% CO_2_.

On the third day, organoids were moved into individual wells of low-adhesion 24-well plates and supplemented with maturation medium (MM). Plates were incubated on an orbital shaker in a 37 °C incubator at 5% CO_2_. Fresh MM was supplied every three to four days.

For further culture on the CERO 3D bioreactor, eight-day-old EBs were moved to CERO tubes containing EM supplemented with 3% MG and incubated in the bioreactor (at 37° C and 5% CO_2_). On the third day, EM was replaced with MM. Fresh MM was supplied every three to four days.

On days 0 and 40, pictures were taken. At day 40 (+/- 2 days), organoids were taken out of the incubator and washed three times in 1x Phosphate-Buffered Saline (PBS). Organoids for proteomic analysis were weighed and snap-frozen on dry ice individually. Organoids for immunofluorescence analysis were fixed in 4% Paraformaldehyde (PFA, Sigma-Aldrich) overnight, then washed three times in 1xPBS and processed as described below.

A detailed protocol for organoid generation in the CERO 3D bioreactor is provided below.

### AI-assisted organoid size measurement

Images of the organoids were captured using an Echo Rebel microscope. Organoid size was quantified by estimating the area occupied by each organoid at defined time points based on image-derived contours. To enable efficient and objective measurements, we used an AI-assisted image segmentation approach based on the Segment Anything Model, which automatically detected candidate organoid shapes. Detected objects were filtered to exclude regions outside the culture dish, shapes of implausible size, or areas with insufficient contrast. All resulting contours were manually reviewed and corrected where necessary in open-source labeling software, ensuring that final organoid boundaries were defined by a human observer. Organoid areas were extracted from the validated contours, with multiple measurements retained per organoid and time point for downstream statistical analysis.

### Quantification of organoid size variability across culture systems

To evaluate whether organoid cultures grown in plates or in tubes differ in size variability, we quantified raw heterogeneity in organoid area across both systems. Organoids originated from eight independent donors (iPSC lines 1–8). In plate cultures, the same organoids were sampled repeatedly over time, whereas in tube cultures, organoids were sampled randomly at each measurement day. For each day, we computed the raw variance and standard deviation (SD) of organoid size separately for plate and tube cultures. A tube/plate variance ratio was then calculated for each day with complete observations. To formally test for systematic differences in heterogeneity between culture systems, we applied a Wilcoxon signed-rank test to the log-transformed variance ratios (log(tube/plate)). A median log-ratio < 0 indicates greater variability in plate cultures. All analyses were performed in R (version 4.4.2).

### Immunofluorescence analysis

Eight to ten PFA-fixed on day 40 (d40) organoids per iPSC line were embedded into optimal cutting temperature (OCT) mounting media (VWR, 361603E) and snap-frozen on crushed dry ice. The embedded organoids were sectioned into 20 μm-thick slices using a cryostat (Leica CM3050S) and applied to Superfrost slides (VWR, 631 − 0108).

The tissue sections were rinsed in demineralized water to remove OCT media, followed by a 3 × 10-minutes wash in washing buffer (1X PBS (VWR, 392–0442) and 0.25% Triton X-100 (Sigma, X100)). Subsequently, slides were blocked in washing buffer with 5% goat serum (Gibco, 16210-064) and 2% Bovine Serum Albumin (BSA, Sigma, A7030) for 1 h at room temperature. The blocking solution was removed, and primary antibody solutions with antibodies targeting neurodevelopmental markers, including PAX6 (1:500, Cell Signaling Technology, D6D9), SOX2 (1:400, Invitrogen 42-6600), TUJ1 (1:500, Stemcell Technologies, 60052), CTIP2 (1:500, Abcam, ab18465), TBR1 (1:2000, Abcam, ab31940), and TBR2 (1:500, Abcam, ab23345), were applied overnight at 4 °C.

Slides were washed 3 × 10-minutes in washing buffer and incubated with secondary antibodies in blocking buffer (Invitrogen, A11034, A11007, and A11032, all diluted at 1:1000) for 2 h at room temperature in the dark. After another 3 × 10-minutes wash in 1X Tris-Buffered saline (Sigma, T5912), including counterstaining with DAPI (Invitrogen, R37606), coverslips were mounted with mounting medium (Agilent Technologies, S3023). Image acquisition was performed with an Olympus FV1000 upright single-photon laser scanning confocal fluorescence microscope.

### Proteomic analysis and Data Independent Acquisition (DIA)

Samples for proteomics analysis were prepared according to the protein aggregation capture protocol (Batth et al. 2019). Briefly, the organoids were lysed in 5% Sodium Dodecyl Sulfate (SDS) with 100 mM 4-(2-hydroxyethyl)-1-piperazineethanesulfonic acid (HEPES) pH 8.0, sonicated until complete transparency of the lysates and centrifuged for 15 min at 16,000 g. The protein concentrations were measured using the Bicinchoninic Acid (BCA) assay kit (Pierce, Thermo Scientific) and 100 µg of protein mixture was mixed with acetonitrile (70% by volume) and 10 µl of Hydrophilic Interaction Liquid Chromatography (HILIC) magnetic beads slurry (ReSyn Biosciences). After 30 min, the beads were washed twice: once with 100% acetonitrile and once with 70% ethanol. The drained beads were resuspended in 100 µl of 100 mM HEPES, pH 8.5. Proteins were reduced with 2 mM Dithiothreitol (DTT) for 30 min at room temperature and alkylated with 11 mM chloroacetamide for 30 min in the dark. Proteins were digested with 1 µg of LysC mixed with 1 µg trypsin for 16 h. Resulted peptide complex mixtures were purified using the Stage Tips setup (McCrory et al. 2022), supplemented with Indexed Retention Time (iRT) standard peptides (Biognosys AG, Switzerland). Peptides were loaded into a home-made fused silica column packed with 1.9 μm Reprosil C18 beads (Dr. Maisch) and analysed by nano Liquid Chromatography-Tandem Mass Spectrometry (nanoLC-MS/MS) using an EASY-nLC 1000 ultra-high-pressure system (Thermo Fisher Scientific) attached online to an Orbitrap Exploris 480 mass spectrometer (Thermo Fisher Scientific) (Boel et al. 2025). Peptides were eluted into the mass spectrometer with a gradient of acetonitrile for 120 min. The Data-Independent Acquisition (DIA) method (Trulsson et al. 2022) was used with the following settings: MS1 scan with a mass range 350–1400 m/z, a normalized Automatic Gain Control (AGC) target set to 300% with 120,000 resolution, and 45 ms of Injection Time (IT). Acquired DIA scans had an AGC target value set at 1000%, *R* = 30,000, and IT to 54 ms. We used a setup of 56 windows, each 12 Da with an overlap of 1 Da. Normalized Higher-energy Collisional Dissociation (HCD) collision energy was set at 28%. Data were acquired in profile mode using positive polarity. Raw file data was handled by Spectronaut (version 17.0.221202.55965) with the Direct DIA approach using the search engine Pulsar with default settings. A reviewed UniProt database (SwissProt from October 2019 of Homo sapiens) with 20,351 entries was used during the search. Oxidation (M) and Acetyl (Protein N-term) were set as variables and carbamidomethyl (C) as fixed peptide modifications. Protein group intensities were normalized using the function of Global Normalization with Median and Data filtering set to Q-value.

### Statistical analysis of proteomic variability

Proteomic data were obtained from six individual cerebral organoids derived from two iPSC lines (iPSC line 9, SK55, and iPSC line 10, WLZ368; *n* = 3 organoids per line), cultured on CERO 3D bioreactor, that differed in size and weight. Individual organoids were drained of excess buffer and weighed. Single organoids were referred to as SK55w42, SK55w64, and SK55w36, weighing 42 µg, 64 µg, and 36 µg respectively, and WLZw52, WLZw40, and WLZw140, weighing 52 µg, 40 µg, and 140 µg respectively. To ensure comparability across samples, only proteins detected in all six organoids were included in downstream analyses, resulting in a final dataset of 6,736 proteins (p) out of 8,590 identified in total. Given the high dimensionality of the dataset (p ≫ n), multivariate differences in protein expression profiles were assessed using Mahalanobis distances approximated by Euclidean distances in the first two principal components derived from principal component analysis (PCA). Within-line and between-line comparisons were performed separately. To identify proteins contributing most to variability, coefficients of variation (CV = σ/µ) were calculated within each line, and linear discriminant analysis (LDA) was used to identify proteins contributing most strongly to between-line separation. All analyses were exploratory, reflecting the limited sample size.

## Results

### Neuroepithelial development and marker expression in cerebral organoids cultured in the CERO 3D bioreactor

**Fig. 1:**
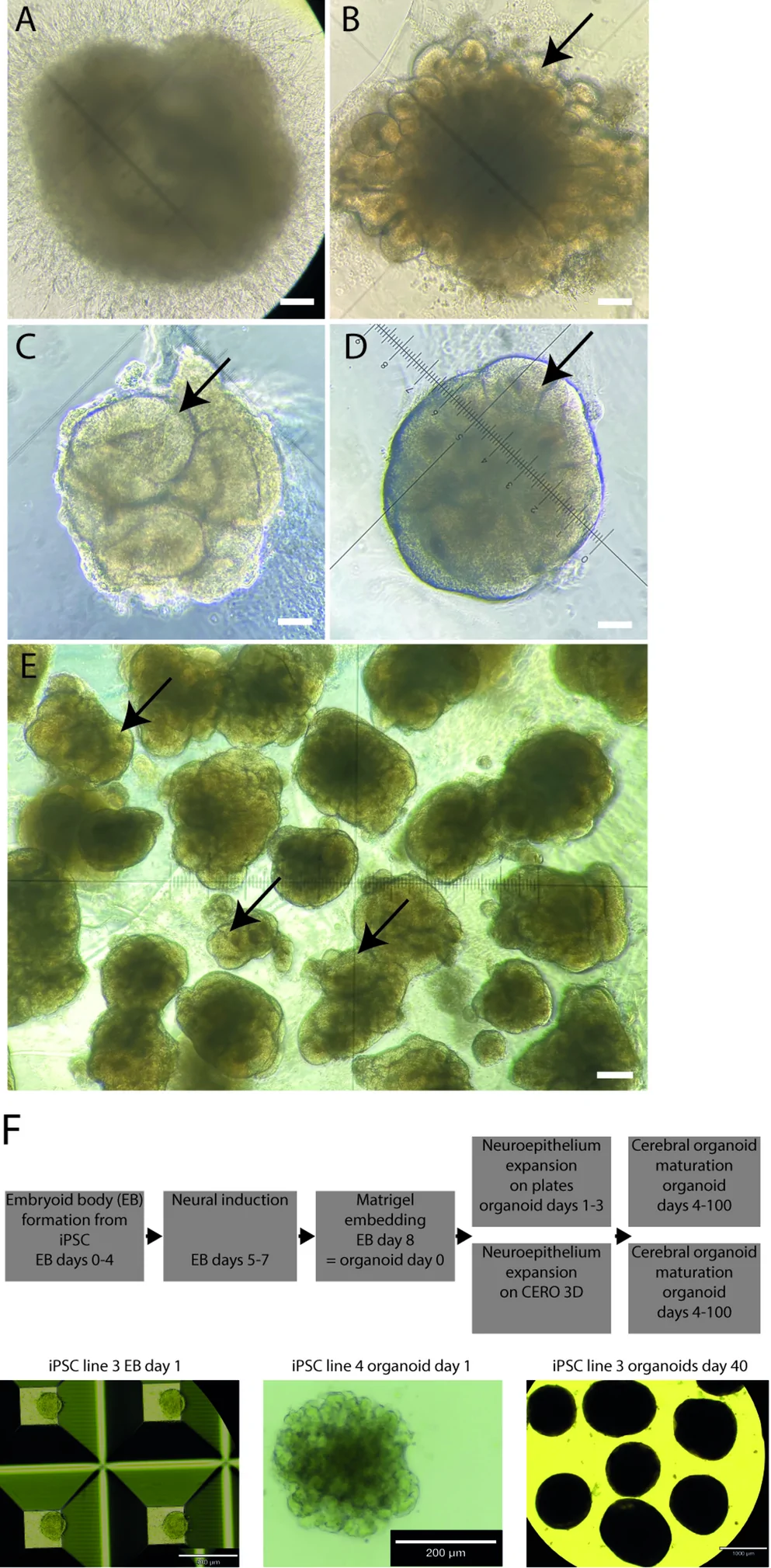
Organoid derivation on plates and in the CERO 3D bioreactor. Arrows indicate neuroepithelium formation manifesting as neural buds.** A**–**D** Light microscopy of day 2 organoids derived from iPSC line 10 (**A**, **C**) and iPSC line 3 (**B**,** B**) (Table 1), cultured either in six-well plates (**A**,** B**) or in the CERO 3D bioreactor (**C**,** D**). Scale bar, 100 μm.** E** Representative image of organoids 7 days after Matrigel. Scale bar, 100 μm.** F** Workflow for organoid production

Based on our previous experiments, we selected two iPSC lines with distinct capacities for neuroepithelial formation to evaluate cerebral organoid generation in the CERO 3D bioreactor. While organoids derived from iPSC line 3 (MR-013, Table 1) readily formed expanded neuroepithelium, manifested as neural buds, under both conventional plate-based conditions and in the bioreactor (Fig. 1B and D), organoids derived from iPSC line 10 (WLZ368, Table 1) previously characterized by poor neural bud formation using traditional methods, readily formed neuroepithelial structures when cultured in the CERO 3D system. Notably, iPSC line 10 derived organoids displayed more robust neural structures in the bioreactor compared with plate-based cultures (Fig. 1A and C).

**Fig. 2:**
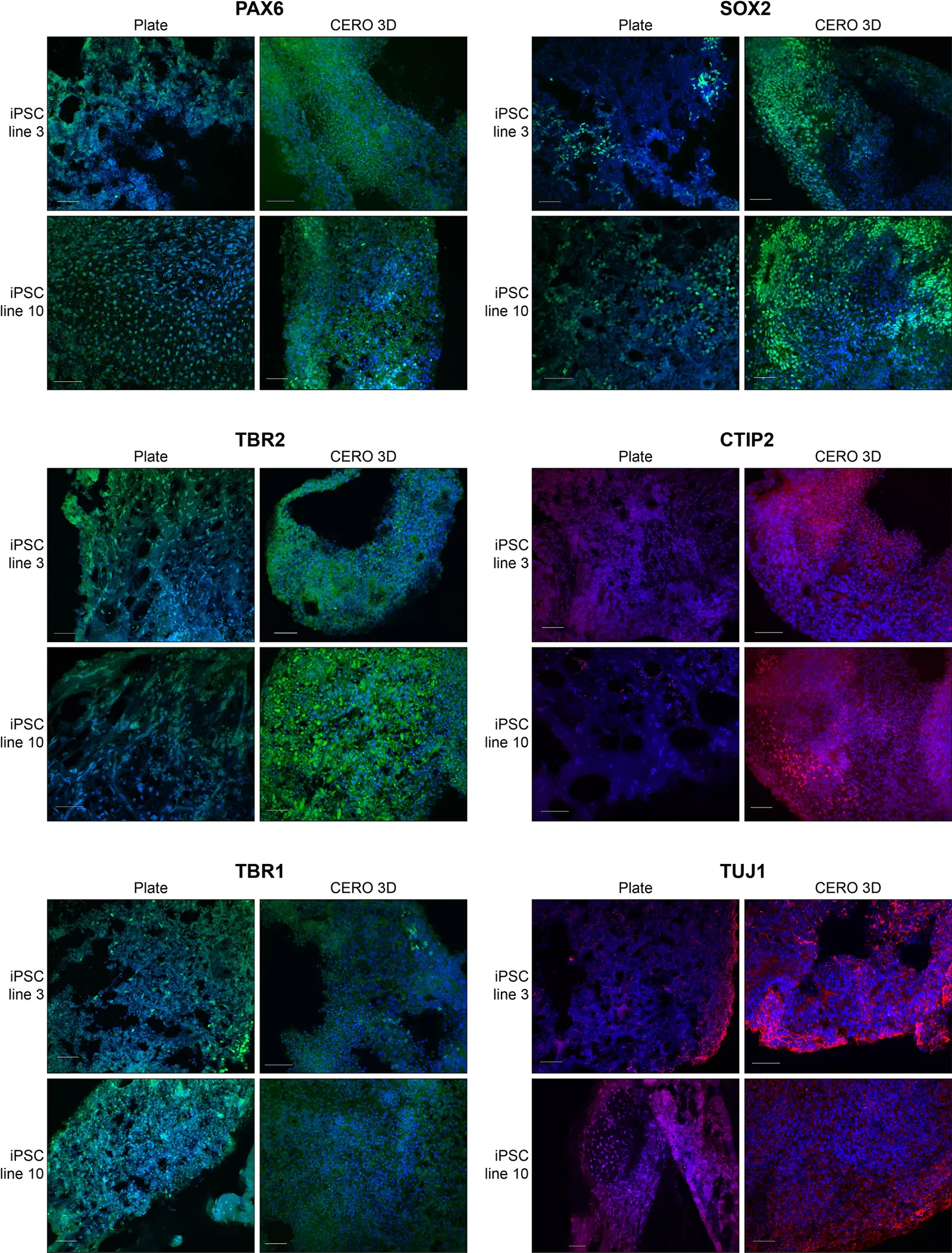
Immunofluorescence characterization of day 40 cerebral organoids generated from iPSC line 10 and iPSC line 3 (Table 1) and cultured either in six-well plates or in the CERO 3D bioreactor. Organoid sections were stained for PAX6 (green), SOX2 (green), TUJ1 (red), CTIP2 (red), TBR1 (green), and TBR2 (green). Nuclei were counterstained with DAPI (blue). Scale bar, 50 μm

To assess cellular differentiation, immunofluorescent labeling was performed on day 40 organoids. Neural progenitor cells were identified using antibodies against PAX6 and SOX2, intermediate progenitor cells were labeled with TBR2, and differentiated neuronal populations were detected using TUJ1, CTIP2, and TBR1. Cerebral organoids generated in the CERO 3D bioreactor from both iPSC lines expressed all neurodevelopmental markers analyzed (Fig. 2).

### Organoids can be produced efficiently in the CERO 3D bioreactor at scale

Using the described procedure, up to 1200 cerebral organoids were generated in a single CERO 3D tube. Medium was exchanged twice per week, with 30 mL per tube per change. Producing a comparable number of organoids using conventional six-well plate cultures require approximately 10 plates, assuming 20 organoids per well, and ~ 360 mL of medium per week (3 mL per well, supplied twice weekly).

### Organoid size-associated and line-associated proteomic variability

We observed variability in both organoid size and morphology, even among organoids cultured in the CERO 3D bioreactor. Since cerebral organoids are stochastic, self-organizing structures, some variability between individual organoids is expected, even when they are generated from the same iPSC line and in the same experiment. This is consistent with previous reports describing heterogeneity in cerebral organoid cultures (Lancaster and Knoblich 2014; Sivitilli et al. 2020). We therefore asked whether organoids from the same line can differ enough at the proteomic level to influence the comparison between different iPSC lines. In this pilot proteomic dataset, we aimed to distinguish two possible sources of variability: variation related to organoid size, and variation related to iPSC line identity.

**Fig. 3:**
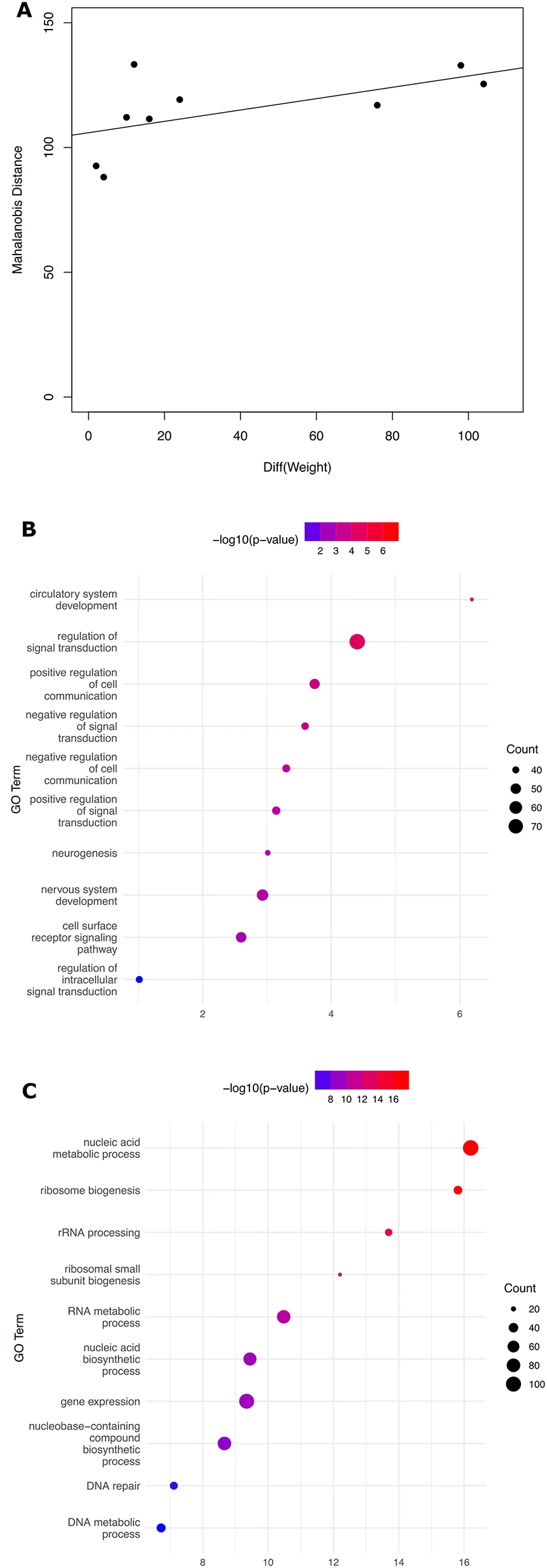
Proteomic analysis of organoid size-associated and iPSC line-associated differences. **A** Relationship between organoid weight differences and proteomic dissimilarity between individual organoids. Each point represents one pairwise comparison. **B** Gene Ontology Biological Process enrichment analysis of proteins associated with organoid size-related variability within the same iPSC line. These terms therefore represent processes differing between organoids of different size from the same genetic background. **C** Gene Ontology Biological Process enrichment analysis of proteins associated with differences between the two iPSC lines. These terms therefore represent line-associated differences and should be interpreted separately from the size-associated analysis shown in panel B. Dot size represents the number of proteins associated with each GO term, and color indicates statistical significance

First, we examined whether organoids of different weights differed in their overall proteomic profiles. For this analysis, comparisons were made within each iPSC line, so that organoids were compared with other organoids derived from the same genetic background. Pairwise differences in organoid weight were plotted against multivariate proteomic dissimilarity, calculated as Mahalanobis distances approximated by Euclidean distances in PCA space (Fig. 3A; Table 2). In both iPSC lines analyzed (line 9, SK55, and line 10, WLZ368), larger differences in organoid weight were associated with larger differences in global protein expression. This suggests that organoid size contributes to proteomic variability within the same iPSC line.

We then asked which biological processes were represented among proteins contributing to this size-associated variability. Importantly, this analysis was performed within each iPSC line and therefore reflects differences between organoids of different size from the same line, not differences between the two iPSC lines. Gene Ontology enrichment analysis showed that differences related to organoid size were mainly associated with developmental and signal-transduction-related processes with the direction of these associations shown in Fig. 3B. This may indicate that organoids of different sizes are not only larger or smaller versions of the same structure but may also differ in aspects of developmental state.

Second, we examined proteomic differences between the two iPSC lines. Between-line differences were observed even when organoids had similar weights, indicating that iPSC line identity contributes to proteomic variability independently of organoid size. Proteins contributing most strongly to the separation between the two lines were enriched for core cellular and biosynthetic processes, including RNA metabolism, ribosome biogenesis, and gene expression (Fig. 3C). These categories therefore reflect line-associated differences rather than size-associated differences.

Together, these results indicate that two distinct sources of proteomic variability were detected in this pilot dataset. Organoid size was associated primarily with differences in developmental and signal-transduction-related processes, whereas iPSC line identity was associated primarily with RNA metabolism, ribosome biogenesis, gene expression, and related core cellular processes. Because the proteomic analysis was based on a limited number of organoids, these findings should be interpreted as exploratory, but they support the importance of controlling organoid size in downstream molecular analyses.

**Table 2:** Pairwise differences in organoid weight and corresponding proteomic distance between individual organoids

	SK55w36	SK55w42	SK55w64
WLZw40	(4, 88.12)	(2, 92.64)	(24, 119.19)
WLZw52	(16, 111.46)	(10, 112.09)	(12, 133.3)
WLZw140	(104, 125.45)	(98, 132.9)	(76, 116.95)

### Organoids produced in CERO 3D are more homogeneous than organoids grown on plates

**Fig. 4:**
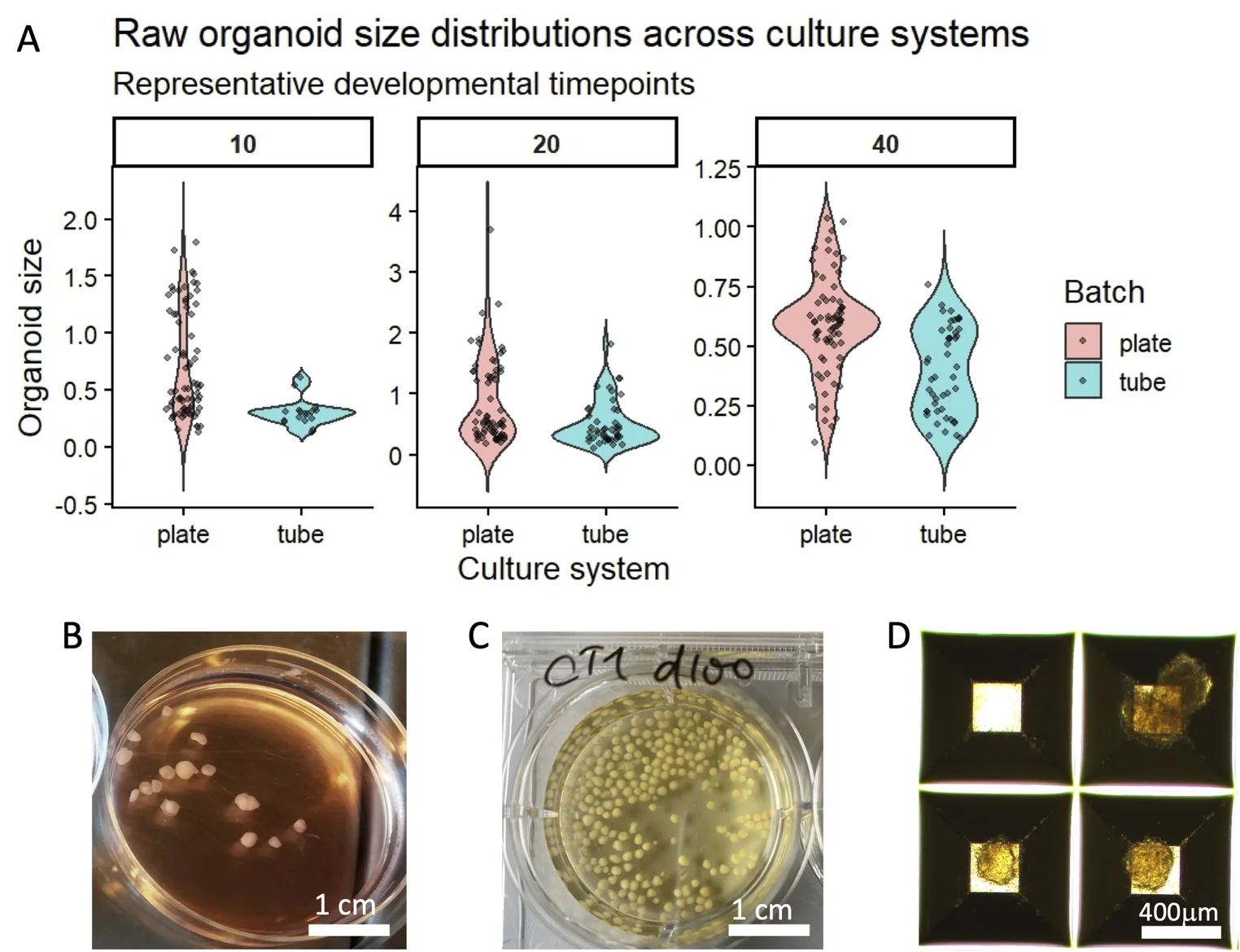
Size variability after organoids generation using the CERO 3D. **A** Raw organoid size distribution (area, mm^2^) of 10-, 20- and 40-day-old organoids using the conventional method (plate) and the CERO 3D bioreactor (tube). Representative images of organoids generated using the conventional method (**B**) and the CERO 3D bioreactor (**C**) at day 100. Embryoid bodies merging on AggreWell plates (**D**)

Size measurements from a total of 1340 organoids produced from iPSC lines 1–8 (Table 1) were analyzed, comprising 991 observations from plate cultures and 349 observations from CERO 3D tube cultures. Across the measurement period (19 discrete measurement days spanning day 0 to day 71 of organoid culture), organoid size variability was markedly higher in plate cultures compared with tube cultures. For nearly all days with complete observations (17 out of 19; number of days used for the heterogeneity test), the raw variance of organoid size in plates exceeded that of tubes, reflected in consistently low tube/plate variance ratios (Fig. 4A). The median variance ratio across all measurement days was 0.289, indicating that plate cultures exhibited approximately 3.5-fold greater size variability than tube cultures. The Wilcoxon signed-rank test confirmed a statistically significant deviation from zero (V = 25, *p* = 0.003), demonstrating that organoids grown in plates were consistently more heterogeneous in size than those cultured in tubes. Overall, these findings show that, at the level of raw observed organoid size distributions, tube cultures yield substantially more homogeneous organoid populations, whereas plate cultures display pronounced variability. Although quantitative size measurements in this study were collected up to day 71, representative images from day 100 are included to illustrate that the observed difference in size homogeneity between culture systems persists at later time points. Representative images of organoids grown to day 100 are shown in Fig. 4B (plates) and Fig. 4C (CERO 3D).

## Discussion

Cerebral organoids provide a powerful model for studying human neurodevelopment, yet their widespread use is limited by substantial variability in size, morphology, and cellular composition, as well as by the labor-intensive nature and limited scalability of conventional culture methods. In this study, we present a refined protocol for cerebral organoid generation using the CERO 3D benchtop bioreactor and demonstrate its advantages in terms of scalability, size homogeneity, neuroepithelial formation, and downstream molecular consistency.

### Sources of organoid size heterogeneity and benefits of large-scale production

Despite improvements achieved with the CERO 3D system, some degree of size heterogeneity remains inherent to cerebral organoid culture. In our setup, size variability arises primarily from two sources: partial merging of EBs during seeding in AggreWell 800 plates (Fig. 4D) and occasional merging of organoids during early culture in bioreactor tubes. These effects are consistent with the stochastic nature of self-organizing 3D systems and have been widely reported in non-directed organoid protocols (Lancaster et al. 2013; Lancaster and Knoblich 2014; Sivitilli et al. 2020). Importantly, the ability to generate organoids at large scale mitigates this limitation by enabling the selection of size-matched organoids for downstream analyses. By selecting organoids of similar size, variability can be reduced at the experimental level, thereby improving the reliability and reproducibility of molecular and functional readouts. Finally, due to substantially larger consumption of culture medium, increased hands-on time with associated contamination risks, and the small-scale nature of plate-based organoid approaches, the CERO 3D system offers clear practical advantages for cost-efficient large-scale organoid production.

### Organoid size as a contributor to proteomic variability

Using an exploratory mass spectrometry-based proteomic approach, we examined whether differences in organoid size are associated with differences in protein expression within the same iPSC line. Although based on a limited number of samples, our analysis indicates that increasing differences in organoid weight are associated with increasing dissimilarity in global proteomic profiles. This observation suggests that size-related variability is not merely a morphological feature but may reflect underlying molecular differences. Notably, proteins contributing most to size-associated variability were enriched for developmental and signaling-related processes, raising the possibility that organoids of different sizes may correspond to distinct neurodevelopmental stages. While these findings are exploratory, they underscore the importance of controlling organoid size in studies aiming to detect subtle molecular phenotypes.

### Improved neuroepithelial formation across iPSC lines

Another major advantage of the CERO 3D bioreactor was the robust formation of neuroepithelial structures across iPSC lines with differing intrinsic capacities for neural induction. While iPSC line 3 (MR-013, Table 1) readily formed neural buds under conventional culture conditions, the iPSC line 10 (WLZ368, Table 1) line consistently failed to do so when grown on plates. In contrast, both lines achieved consistently high neuroepithelial formation when cultured in the CERO 3D system. These results suggest that the near-microgravity environment and continuous suspension provided by the bioreactor enhance early neurodevelopmental processes, potentially by improving nutrient distribution and reducing mechanical stress. Importantly, this increased robustness across cell lines addresses a key limitation of cerebral organoid technology and supports broader applicability of the protocol.

### Reduced labor, medium consumption, and experimental variability

Conventional organoid culture requires extensive manual handling, including repeated transfers between plates and individual embedding in extracellular matrix, which limits throughput and increases the risk of variability and contamination. In contrast, the CERO 3D bioreactor enables the generation and maintenance of up to 1200 organoids per tube with substantially reduced hands-on time and medium consumption. This reduction in labor and material costs not only improves efficiency but also contributes to more stable and consistent culture conditions, which are essential for reproducible large-scale studies and screening applications.

## Limitations of the study

Several limitations of the present study should be acknowledged. The proteomic analyses were performed on a small number of organoids derived from two iPSC lines and should therefore be interpreted as proof-of-concept rather than definitive evidence of causal relationships. In addition, precise quantification of organoid number during long-term culture in bioreactor tubes remains technically challenging. Finally, the CERO 3D system requires specialized equipment and maintenance, which may limit accessibility for some laboratories.

## Implications and future applications

Despite these limitations, the scalability and improved homogeneity achieved with the CERO 3D bioreactor represent a significant advancement for cerebral organoid technology. The ability to generate large numbers of organoids and select size-matched populations enhances statistical power and reproducibility, which is particularly important for applications such as drug screening, toxicology, and comparative molecular analyses. Moreover, the near-microgravity environment of the bioreactor offers opportunities to investigate the role of physical forces in neurodevelopment, complementing emerging studies using microgravity platforms. In the future, this protocol may facilitate large-scale studies of patient-derived organoids, co-culture systems, and personalized medicine approaches.

In summary, this study presents a refined and scalable protocol for cerebral organoid generation using the CERO 3D benchtop bioreactor. By enabling the production of large numbers of organoids with improved size homogeneity and robust neuroepithelial formation across iPSC lines, this approach addresses key technical limitations of conventional plate-based methods. Our exploratory proteomic analyses further highlight the relevance of organoid size as a contributor to molecular variability, underscoring the importance of size control in downstream applications. Together, these findings support the use of the CERO 3D bioreactor as a practical platform for reproducible, high-throughput cerebral organoid culture in neurodevelopmental research.

## Data Availability

The data supporting the findings of this study are available from the corresponding author upon reasonable request.

## Detailed protocol for the culture of cerebral organoids in CERO 3D bioreactor

Reagents needed:

STEMdiff™ Cerebral Organoid Kit (STEMCELL Technologies, 08570).

Matrigel (Corning, 354277).

Penicillin/Streptomycin (Sigma-Aldrich, P4333).

Gentle Cell Dissociation Reagent (STEMCELL Technologies, 100–0485).

Anti-Adherence Rinsing Solution (STEMCELL Technologies, 07010).

Revitacell (Gibco, A26445-01).

AggreWell 800 plates (STEMCELL Technologies, 34811).

CERO tubes (OLS OMNI Life Science, 195280).

CERO 3D bioreactor (OLS OMNI Life Science, 2800000).

**EB Generation Protocol**:

**1. Cell Preparation**:

- Grow induced Pluripotent Stem Cells (iPSCs) to 60–80% confluency.
- Remove the medium and rinse the well with 2 ml of phosphate-buffered saline (PBS).

**2. Cell Dissociation**:

- Add 1 ml of Gentle Cell Dissociation Reagent (GCDR) per well and incubate at 37 °C for 10 min.
- Add 2 ml of Seeding Medium (SM) per well, pipet vigorously to dissociate the cells, and transfer the cells to a 15 ml tube.
- Centrifuge the cells at 300 g for 3 min.
- Resuspend the cell pellet in 2 ml of SM.
- Count the cells.

**3. Coating AggreWells**:

- Coat the required number of AggreWells with Anti-Adherence Rinsing Solution (AARS).
  - Add 0.5 ml of AARS per well, rock gently to coat the well walls, and inspect visually for air bubbles. Remove any bubbles by pipetting.
  - Remove AARS and rinse each well with 0.5 ml of SM once.

**4. Cell Seeding**:

- Calculate the volume corresponding to 900,000 cells per well of a 24-well AggreWell 800 plate, multiplied by the number of wells to be seeded. For a typical experiment, 1200 organoids will be placed in each CERO tube, obtained from culturing EBs in four AggreWell 800 wells.
- Supplement the cell suspension with SM to a total volume of 1 ml per well and mix thoroughly.
- Add 1 ml of the cell suspension to each well.

**5. Incubation**:

- Place the AggreWell plate in an incubator and do not disturb until the next day.
- Handle the plate with extreme care to avoid disturbing the embryoid bodies (EBs) and prevent them from floating into neighbouring wells.

**6. Medium Exchange**:

- On day 1, add 1 ml of Formation Medium (FM) to each well as slowly as possible along the well wall using the gravity setting on the pipette to avoid disturbing the EBs.
- On days 2, 3, and 4, remove 1 ml of FM and add 1 ml of fresh FM.
- On day 5, replace FM with Induction Medium (IM) by removing 1.5 ml of FM and adding 1.5 ml of IM, repeating as necessary to replace as much FM as possible.
- On days 6 and 7, remove 1 ml of IM and add 1 ml of fresh IM.

**Organoid Generation**:

**1. Preparation**:

On day 8, supplementation of EBs with 3% Matrigel (MG) and transfer to the CERO 3D bioreactor marks organoid day 0.

- Program the CERO 3D bioreactor with a 2-second rotation in each direction at 80 rpm, with a 1-second pause.
- Thaw sufficient MG to achieve a final concentration of 3% and keep on ice.
- Prepare 5 ml of Expansion Medium (EM) per CERO tube and place in a 50 ml tube on ice.

**2. Transfer EBs**:

- Remove most of the IM from the wells containing EBs and add 1 ml of EM.
- Gently pipet 2–3 times and transfer the suspension to the CERO tube.
- Rinse the well with 1 ml of fresh EM and transfer to the CERO tube, repeating once more.
- Add 10 ml of EM to the CERO tube for a total volume of 13 ml.

**3. MG Supplementation**:

- To the ice-cold EM, add MG (0.6 ml MG to 5 ml EM per CERO tube) and mix thoroughly by pipetting 4–5 times.
- Add 5 ml of the MG/EM mixture to the CERO tube containing EBs and mix gently by pipetting 2–3 times.
- Place the tube on the CERO 3D bioreactor and start the program.

**4. Initial Handling**:

- After 20–30 min, remove the tubes from the bioreactor and gently pipet 2–3 times to break MG clots, preventing EBs from sticking to clots and merging, which leads to size heterogeneity.

**5. Medium Exchange**:

- On day 3, remove EM and replace it with Maturation Medium (MM).
- Feed with fresh MM every 2–4 days, depending on the number of organoids in the CERO tube.
  - To feed, remove the tube from the bioreactor and allow organoids to settle for 30–60 s.
  - Remove as much spent medium as possible, leaving approximately 5 ml to avoid aspirating organoids, and add fresh medium.
  - Typically, with 1200 organoids per tube, feed every 3–4 days, maintaining a volume of 40–45 ml.

**General Precautions**:

- Maintain sterility throughout the process. Contamination of a tube will compromise the entire experiment.

## Abbreviations

AARS: Anti-adherence rinsing solution
AGC: Automatic gain control
BCA: Bicinchoninic acid
BSA: Bovine serum albumin
CV: Coefficients of variation
DIA: Data-independent acquisition
DTT: Dithiothreitol
EB: Embryoid body
EM: Expansion medium
FM: Formation medium
GCDR: Gentle cell dissociation reagent
GO: Gene ontology
HCD: Higher-energy collisional dissociation
HEPES: 4-(2-Hydroxyethyl)-1-piperazineethanesulfonic acid
HILIC: Hydrophilic interaction liquid chromatography
IM: Induction medium
iPSC: Induced pluripotent stem cells
iRT: Indexed retention time
IT: Injection time
LDA: Linear discrimination analysis
MG: Matrigel
MM: Maturation medium
nanoLC-MS/MS: Nano liquid chromatography-tandem mass spectrometry
OCT: Optimal cutting temperature
PBMC: Peripheral blood mononuclear cells
PBS: Phosphate-buffered saline
PCA: Principal component analysis
PFA: Paraformaldehyde
SDS: Sodium dodecyl sulfate
SM: Seeding medium
TBS: Tris-buffered saline
